# Supplementary material for: NREM sleep oscillations and their relations with sleep-dependent memory consolidation in early course psychosis and first-degree relatives
Source: bioRxiv. 2023 Nov 2:2023.10.30.564703. Preprint. [Version 1] doi: 10.1101/2023.10.30.564703 (PMC10634996; doi:10.1101/2023.10.30.564703)
Supplement: 1 [file NIHPP2023.10.30.564703v1-supplement-1.pdf]

bioRxiv preprint doi: https://doi.org/10.1101/2023.10.30.564703; this version posted November 2, 2023. The copyright holder for this preprint (which was not certified by peer review) is the author/funder, who has granted bioRxiv a license to display the preprint in perpetuity. It is made available under aCC-BY 4.0 International license.

Supplementary results

Correlations between N3 coupled spindles and WPT consolidation

A significant main effect was observed when this analysis was restricted to just coupled spindles during N3 sleep (14 electrodes, F_{sum} = 71.22, p = .041). This main effect was superseded by a significant spindle * group interaction (14 electrodes, F_{sum} = 61.83, p = .046; **Supplementary Figure 3**), reflecting that N3 coupled spindle density was positively associated with the overnight retention of word pairs in the EC group only (r = .66, p = .019; ecSZ: r = .89, p = .02; ecNSZ: r = .64, p = .09; HC: r = .03, p = .88; FHR: r = .25, p = .32). Coupled spindle density was a significantly better predictor of WPT consolidation (BIC: M = 465.78, SD = 3.46) than uncoupled spindle density (BIC: M = 467.97, SD = 3.25), t (57) = 7.43, p < .001, d = 0.65. There was no association between consolidation of the MST and consolidation of the WPT (r = .11, p = .39).

Table S1. Early course patient diagnosis and medications

Patient	Diagnosis	Antipsychotic	Chlorpromazine equivalent dosage (mg)	Antidepressant	Benzodiazepine	Other
1	Schizophrenia		0			
2	Schizophrenia		0			
3	Schizophrenia	Lurasidone (160mg)	800	Bupropion (300mg)		
4	Schizophrenia	Olanzapine (10mg); Perphenazine (10mg)	325			Benztropine (10mg)
5	Schizophrenia		0			
6	Schizophrenia		0			
7	Schizophrenia	Clozapine[1] Aripiprazole[1]				Lithium; Metformin; Larissa; Calcium
8	Schizoaffective disorder, depressive type	Aripiprazole (15mg)	200			Levothyroxine (75 µg)
9	Schizoaffective disorder, depressive type	Aripiprazole (15mg)	200	Fluoxetine (10mg)		Lamotrigine (200mg)
10	Schizoaffective disorder, depressive type	Perphenazine (8mg)	100			Benztropine (1mg)
11	Schizoaffective disorder, bipolar type	Aripiprazole (15mg)	200	Duloxetine (60mg)		Lithium (1200mg)
12	Schizoaffective disorder, bipolar type	Ziprasidone (60mg)	100			Lithium (750mg)
13	Psychosis NOS	Aripiprazole (5mg)	75			Cefalexin (500mg); Melatonin (5mg)
14	Bipolar disorder	Olanzapine (10mg)	200	Fluoxetine (5mg)		Lamotrigine (10mg); Benztropine (1mg)
15	Bipolar disorder	Quetiapine (400mg)	525			Zolpidem (7.5mg); Sodium valproate (1000mg);
16	Bipolar disorder		0			Lamotrigine (100mg)
17	Bipolar disorder		0		Clonazepam (250 µg)	Progesterone; Lamotrigine (100mg); Spironolactone

Note. [1] No dosage information available

Table S2. Sleep architecture for each night

	Visit 1						Visit 2					
	Baseline			Learning			Baseline			Learning		
	HC	FHR	EC	HC	FHR	EC	HC	FHR	EC	HC	FHR	EC
Total sleep time (min)	504 (84)	476 (86)	501 (54)	494 (92)	504 (71)	509 (61)	515 (66)	463 (109)	507 (53)	519 (44)	497 (83)	513 (54)
Sleep onset latency (min)	26 (17)	28 (25)	20 (23)	27 (43)	38 (44)	18 (11)	22 (39)	36 (47)	24 (29)	22 (20)	31 (27)	17 (9)
Sleep efficiency (%)	86 (7)	83 (9)	85 (9)	86 (9)	84 (11)	86 (9)	86 (10)	81 (13)	84 (10)	86 (7)	83 (13)	85 (8)
Wake after sleep onset (min)	51 (39)	62 (41)	62 (52)	48 (32)	46 (35)	63 (48)	50 (49)	62 (56)	71 (53)	59 (44)	57 (61)	62 (42)
N1 (min)	28 (21)	29 (21)	30 (28)	30 (26)	26 (21)	35 (43)	31 (22)	26 (21)	27 (23)	34 (25)	31 (30)	46 (61)
N2 (min)	266 (63)	259 (71)	265 (65)	249 (62)	255 (59)	255 (49)	270 (50)	237 (61)	262 (60)	266 (39)	248 (63)	255 (56)
N3 (min)	99 (30)	106 (44)	116 (37)	106 (37)	114 (43)	125 (57)	104 (27)	105 (38)	124 (53)	99 (30)	122 (61)	119 (42)
REM (min)	112 (30)	83 (37)	90 (34)	109 (40)	109 (33)	94 (47)	110 (32)	95 (35)	94 (51)	121 (28)	96 (37)	93 (51)
N1 (% of TST)	5.7 (4.4)	6 (3.9)	6.1 (5.8)	6.2 (5.1)	5.1 (3.9)	7.3 (8.9)	6 (4.3)	5.7 (4.5)	5.6 (5)	6.6 (5)	6.1 (5.2)	9.2 (12.4)
N2 (% of TST)	52.5 (7.6)	54.0 (9.3)	52.6 (10.7)	50.2 (7.1)	50.5 (8.6)	50.5 (9.1)	52.4 (6.2)	51.5 (5.9)	51.6 (10.6)	51.1 (6.1)	50.0 (9.4)	49.9 (10.4)
N3 (% of TST)	20.2 (6.7)	22.9 (9.6)	23.2 (7.2)	22.1 (7.8)	23 (8.8)	24.2 (9.8)	20.5 (5.7)	23.1 (7)	24.4 (9.5)	19.1 (5.3)	24.9 (11.6)	13.1 (8.2)
REM (% of TST)	21.7 (4.6)	17.1 (6.6)	18.1 (7.1)	21.5 (6)	21.5 (5.1)	18.1 (8)	21 (4.8)	19.8 (4.5)	18.5 (7.5)	23.2 (4.6)	19 (5.8)	17.7 (8.7)

Note. Sleep efficiency was calculated as the percentage of the sleep period (time between sleep onset and final awakening) spent asleep. All values displayed are mean (standard deviation). TST = total sleep time, HC = Healthy controls, FHR = Familial high risk, EC = Early course psychosis.

Table S3. Main effects of group, and interactions involving group, on sleep parameters

	Group main effect			Group * Visit			Group * Session			Group * Visit * Session		
	#	F_{sum}	*p*	#	F_{sum}	*p*	#	F_{sum}	*p*	#	F_{sum}	*p*
**Power spectral density**												
.3-1.25Hz PSD	-	-	-	-	-	-	-	-	-	1	3.16	.397
1.25-4Hz PSD	-	-	-	1	4.43	.362	-	-	-	-	-	-
4-8Hz PSD	-	-	-	1	3.05	.401	4	14.44	.177	-	-	-
8-12Hz PSD	-	-	-	1	3.31	.502	-	-	-	-	-	-
12-15Hz PSD	**37**	**153.26**	**.007**	5	18.67	.088	-	-	-	-	-	-
				6	23.30	.059						
15-30Hz PSD	-	-	-	-	-	-	-	-	-	-	-	-
**Sleep spindles**												
Spindle density	**52**	**249.12**	**.009**	-	-	-	-	-	-	-	-	-
Spindle amplitude	**53**	**309.80**	**.002**	15	59.77	.082	-	-	-	-	-	-
**Slow oscillations**												
SO density	-	-	-	-	-	-	-	-	-	-	-	-
SO amplitude	-	-	-	1	3.19	.420	-	-	-	-	-	-
				1	3.44	.380						
**SO-spindle coupling**												
Coupled spindle count	-	-	-	-	-	-	-	-	-	-	-	-
Coupled spindle density	-	-	-	-	-	-	-	-	-	-	-	-
% coupled spindles	5	18.69	.075	-	-	-	-	-	-	-	-	-
	11	42.82	.436									
	10	36.16	.438									
	7	25.34	.478									
Spindle coupling phase	2	7.55	.631	-	-	-	-	-	-	n/a	n/a	n/a
	2	8.25	.624									
	2	7.06	.643									
	1	3.49	.751									
Spindle coupling consistency	**56**	**277.61**	**.005**	1	3.29	.448	-	-	-	-	-	-

Note: # = the number of channels that formed a cluster, "-" indicates that no cluster was formed. All tests were linear mixed effects models, with the exception of spindle coupling phase, which was assessed via a series of 2-way circular ANOVAs

bioRxiv preprint doi: https://doi.org/10.1101/2023.10.30.564703; this version posted November 2, 2023. The copyright holder for this preprint (which was not certified by peer review) is the author/funder, who has granted bioRxiv a license to display the preprint in perpetuity. It is made available under aCC-BY 4.0 International license.

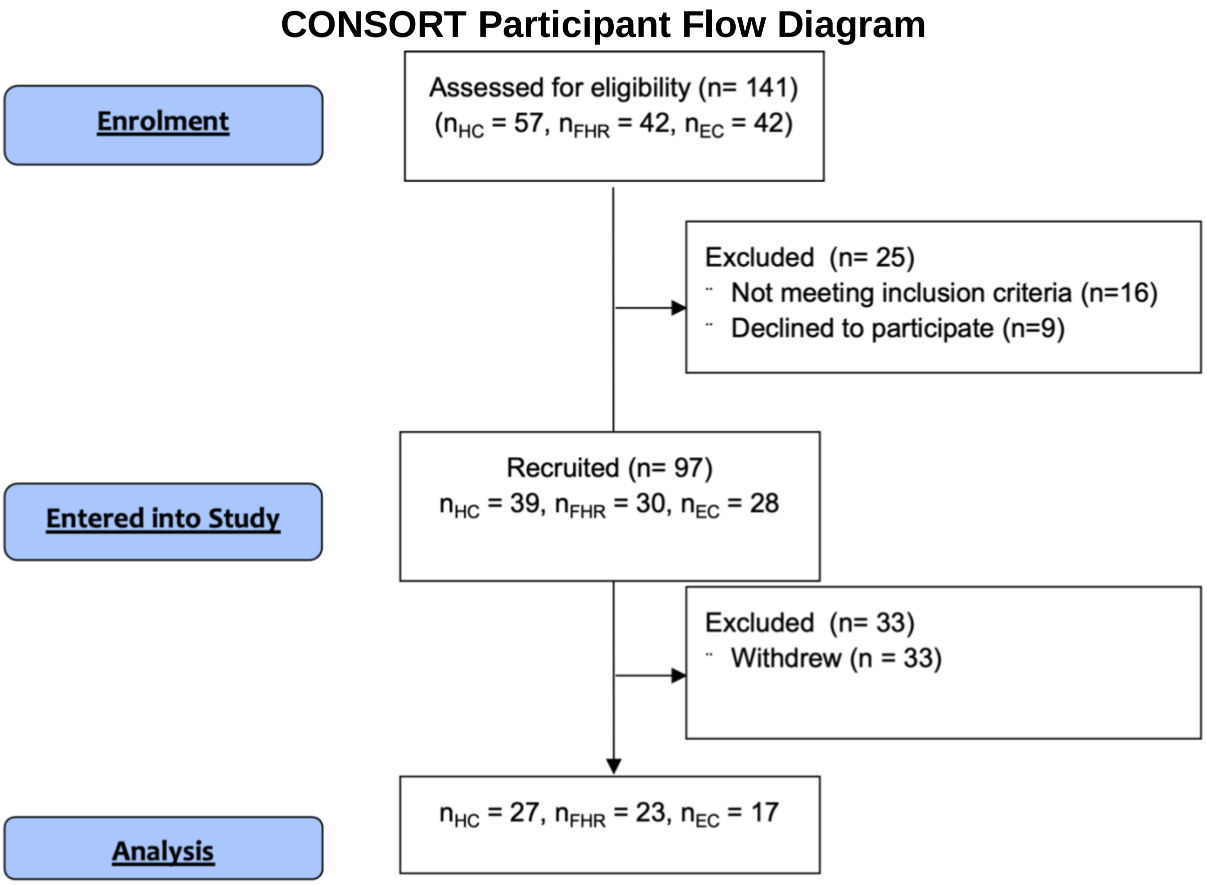

Figure S1. Participant enrolment. HC = Healthy control, FHR = Familial high risk relative, EC = Early course psychosis.

bioRxiv preprint doi: https://doi.org/10.1101/2023.10.30.564703; this version posted November 2, 2023. The copyright holder for this preprint (which was not certified by peer review) is the author/funder, who has granted bioRxiv a license to display the preprint in perpetuity. It is made available under aCC-BY 4.0 International license.

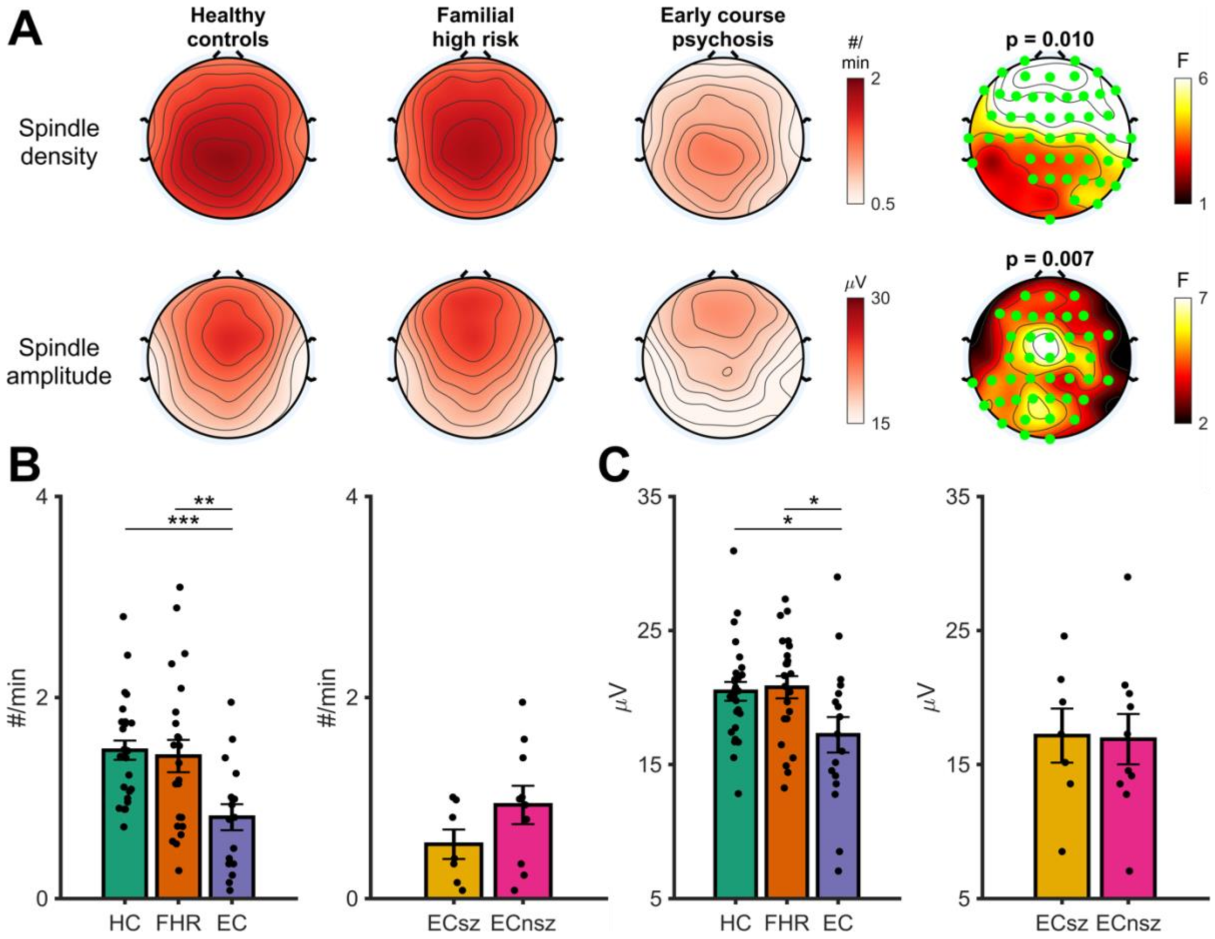

Figure S2. Group differences in N3 sleep spindles. . **A** - Topographies showing the main effect of group on N3 spindle density (Row 1) and amplitude (Row 2). N3 spindle topographies (averaged across all four nights) are shown for the three groups separately. The right-hand plot shows F values at each electrode for the main effect of group. Significant electrodes (cluster-corrected) are highlighted in green. Cluster *p*-value displayed above plot. **B** - Pairwise tests for the main effect of group on N3 spindle density with spindle density averaged over significant electrodes in the cluster highlighted in A. Left hand plot shows differences between the three groups. Right hand plot shows differences between schizophrenia and non-schizophrenia psychosis patients. **C** - Same as B, but for N3 spindle amplitude. HC = Healthy controls, FHR = First degree relatives, EC = Early course psychosis, ECsz = Early course schizophrenia, ECnsz = Early course non-schizophrenia psychosis. Error bars indicate the standard error. *** = $p < .001$, ** = $p < .01$, * = $p < .05$ from pairwise estimated marginal means tests.

bioRxiv preprint doi: https://doi.org/10.1101/2023.10.30.564703; this version posted November 2, 2023. The copyright holder for this preprint (which was not certified by peer review) is the author/funder, who has granted bioRxiv a license to display the preprint in perpetuity. It is made available under aCC-BY 4.0 International license.

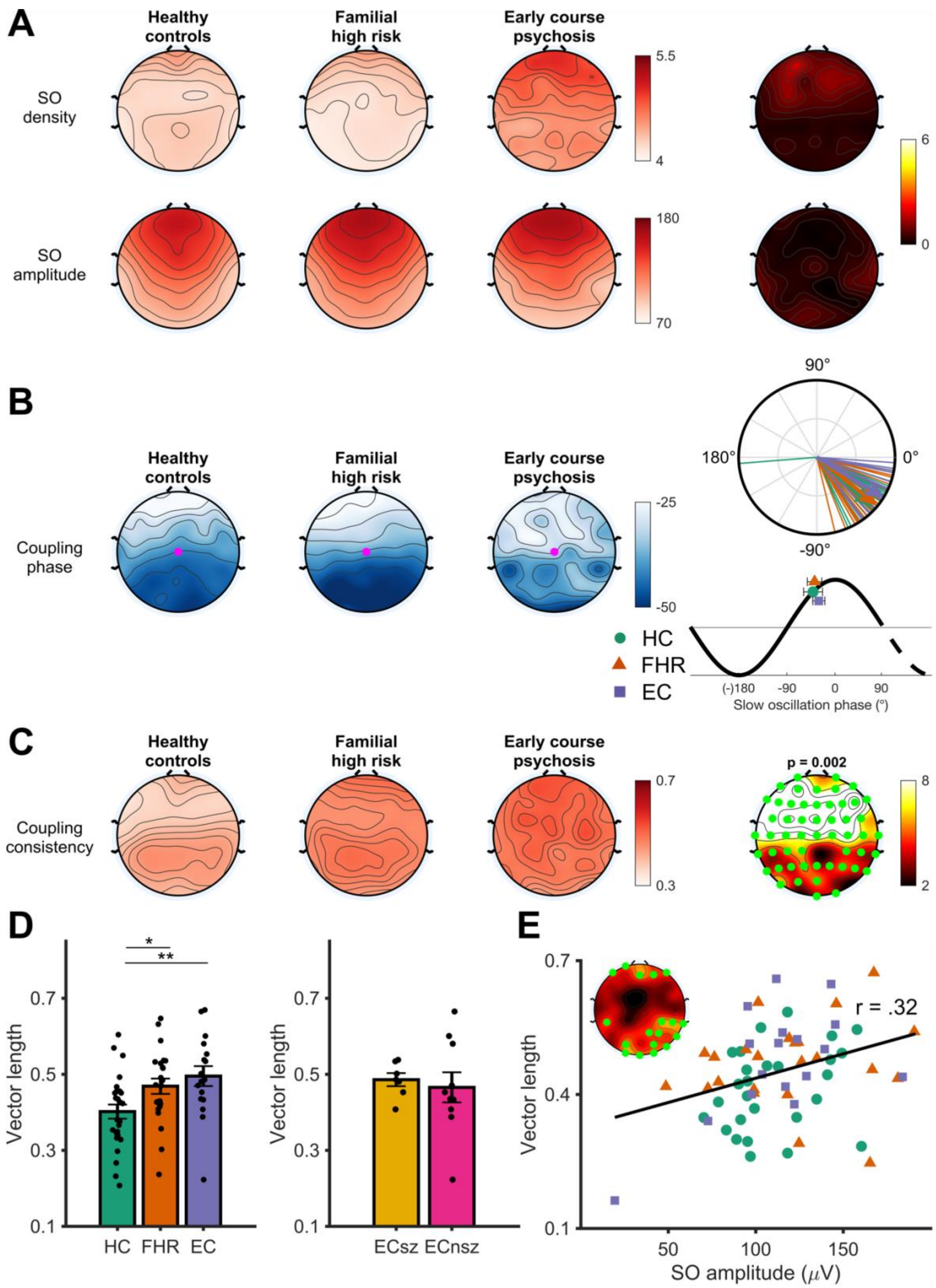

Figure S3. N3 slow oscillation spindle coupling. **A -** Slow oscillation density (top row) and peak-to-peak amplitude (bottom row) in each group, averaged across the four nights. Right most topoplots display F values for the main effect of group. **B -** Preferred coupling phase of spindles to slow oscillations (SOs) in each group, averaged across the four nights. Right: circular phase plot displaying phase distributions across participants for each group at electrode Cz (highlighted electrode in topography). Each line indicates the preferred coupling phase of individual participants. The direction of the arrow indicates the average phase across participants, separately for each group, with the length of the arrow indicating

bioRxiv preprint doi: https://doi.org/10.1101/2023.10.30.564703; this version posted November 2, 2023. The copyright holder for this preprint (which was not certified by peer review) is the author/funder, who has granted bioRxiv a license to display the preprint in perpetuity. It is made available under aCC-BY 4.0 International license.

the coupling consistency across participants. A coupling of phase of 0° indicates preferential coupling of spindles at the positive peak of the slow oscillation. A coupling phase of 180° indicates preferential spindle coupling at the negative trough of the slow oscillation. Mapping of SO phase to topographical and circular plots illustrated underneath circular phase plot **C** - Topographies showing the main effect of group on N3 coupling consistency. Coupling consistency (measured as the mean vector length) topographies (averaged across all four nights) are shown for the three groups separately. The right hand plot shows F values at each electrode for the main effect of group. Significant electrodes (cluster-corrected) are highlighted in pink. Cluster *p* value displayed above plot. **D** - Pairwise tests for the main effect of group on N3 coupling consistency, with coupling consistency averaged over significant electrodes in cluster. Left hand plot shows group differences between the three groups. Right hand plot shows differences between schizophrenia and non-schizophrenia psychosis patients. HC = Healthy controls, FHR = First degree relatives, EC = Early course psychosis, ECsz = Early course schizophrenia, ECnsz = Early course non-schizophrenia psychosis. Error bars indicate the standard error. ** = $p < .01$, * = $p < .05$ from pairwise estimated marginal means tests. **E** – Robust linear regression between slow oscillation amplitude and spindle coupling consistency. Insert shows significant electrodes.

bioRxiv preprint doi: https://doi.org/10.1101/2023.10.30.564703; this version posted November 2, 2023. The copyright holder for this preprint (which was not certified by peer review) is the author/funder, who has granted bioRxiv a license to display the preprint in perpetuity. It is made available under aCC-BY 4.0 International license.

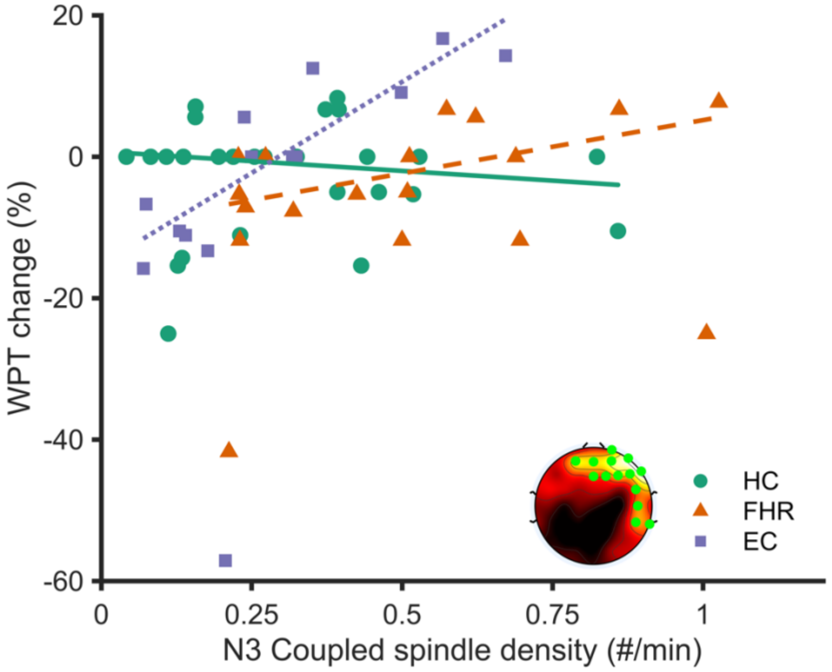

Figure S4. Robust linear regression showing the relationship between coupled N3 sleep spindles and overnight change in word pair memory. Insert shows significant electrodes in the spindle * group interaction (cluster corrected).
